# Stochastic intracellular calcium dynamics show preserved structures identified by deep learning classification

**DOI:** 10.1371/journal.pcbi.1014240

**Published:** 2026-04-29

**Authors:** Jaesung Choi, Athokpam Langlen Chanu, Shakul Awasthi

**Affiliations:** 1 Center for Artificial Intelligence and Natural Sciences, Korea Institute for Advanced Study (KIAS), Seoul, Republic of Korea; 2 Asia Pacific Center for Theoretical Physics (APCTP), Pohang, Republic of Korea; 3 Department of Physics, Pohang University of Science and Technology (POSTECH), Pohang, Republic of Korea; 4 School of Physics, Korea Institute for Advanced Study (KIAS), Seoul, Republic of Korea; Duke University, UNITED STATES OF AMERICA

## Abstract

Intracellular calcium ions (Ca^2+^) exhibit diverse dynamical behaviors linked with cellular physiological states related to health and disease. While deterministic models predict how biochemical parameters create distinct dynamical regimes — steady states, oscillations, bursting, chaos, and multiple periodicity — real biological systems are inherently stochastic due to finite molecular populations. Previous studies using conventional statistical measures demonstrated that increasing intrinsic fluctuations render these dynamical states increasingly indistinguishable, particularly for chaotic and multiple-periodicity patterns. This raises whether parameter-dependent organizational principles persist under realistic noise levels to remain biologically meaningful and computationally detectable. We address this using a large-kernel convolutional neural network (LKCNN) designed to capture global dynamical features across noise levels. Using chemical Langevin equations to generate synthetic training data with realistic intrinsic fluctuations, the LKCNN achieves ~90% accuracy in classifying eight distinct dynamical states despite noise levels that visually obscure distinctions. Validation with experimental Ca^2+^ data from pancreatic β-cells as well as other cells, including WT-HEK293, STIM-KO, and ORAI TKO, achieves 96.8% accuracy, confirming generalizability beyond synthetic datasets, substantially outperforming conventional baselines (Support Vector Machine and Random Forest), which achieve only 54.0% and 51.6% accuracy respectively on the same experimental data. These results demonstrate that deterministic organizational signatures persist through realistic biological noise, suggesting parameter-dependent dynamical structures represent robust principles governing cellular function. Our findings establish that sophisticated pattern recognition can bridge theoretical deterministic dynamics and noisy biological reality, offering a framework for extracting meaningful dynamical information from inherently stochastic oscillatory biological processes.

## Author summary

Cells communicate through calcium signaling, and the rhythms of these signals are deeply connected to physiological states that influence health and disease. Deterministic models predict that these dynamics fall into distinct regimes such as steady states, oscillations, bursting, chaos, and multiple periodicity. However, the intrinsic noise of living systems often obscures these patterns, raising the question of whether they remain meaningful under biologically realistic conditions. To address this, we developed a large kernel convolutional neural network (LKCNN) designed to capture global dynamical features even in the presence of high noise. Trained on synthetic time series data generated with chemical Langevin equations, the model achieved approximately 90 percent accuracy in classifying eight calcium dynamical states that appear visually indistinguishable under noise. When validated with experimental data from pancreatic β-cells as well as other cell types, including WT HEK293, STIM KO, and ORAI TKO, the framework reached 96.8% accuracy, confirming its generalizability beyond simulated datasets. These findings demonstrate that theoretical organizational principles do persist beneath biological variability and that they are computationally detectable. More broadly, this work establishes a pathway for uncovering robust dynamical signatures in inherently stochastic cellular processes, offering new opportunities for decoding hidden determinants of cellular health.

## Introduction

Patterns are ubiquitous in nature across multiple scales, manifesting in phenomena as diverse as the intricate morphologies of supernovae and galaxies [1,2], atmospheric turbulence in Earth’s climate system, geophysical flows, and plasma [3], the collective motion in flocking birds and humans [4,5], and the rhythmic activity within biological cells [6]. At the cellular scale, calcium ions (Ca^2+^), which serve as crucial intracellular messengers, display rhythmic patterns in their cytosolic concentration when the cell is stimulated by an extracellular agonist [7,8]. These rhythmic patterns are termed as intracellular Ca^2+^ oscillations, and are observed across various cell types, including pancreatic cells [9,10], hepatocytes [11], muscle cells [12,13], and neurons [14]. Intracellular Ca^2+^ oscillations play crucial roles not only in signal transduction inside the cell [15,16] but also in regulating various physiological processes, including gene expression [17], cell proliferation [18], and neuronal differentiation [19].

Experimental studies observe complex temporal patterns of intracellular Ca^2+^ oscillations. To explain these complex patterns, several mathematical models have been developed, starting from simple minimal models [20] to complex inositol trisphosphate (InsP_3_) gated models [21–23]. In particular, Houart *et al.* [24] developed a deterministic model based on the non-linear feedback mechanism of the Ca^2+^-induced Ca^2+^ release (CICR) mechanism [25], a process prevalent in various cell types, including hepatocytes [26] and cardiac [27] cells. In the CICR mechanism, the release of Ca^2+^ from intracellular stores into the cytosol is activated by both InsP_3_ and cytosolic Ca^2+^ itself, forming an autocatalytic feedback loop. This mechanism gives rise to a variety of complex dynamical behaviors, including periodic spikes and bursting in the Ca^2+^ oscillation patterns. Theoretical modeling and experimental observations of these complex Ca^2+^ oscillations are important lines of investigation for a deeper understanding of real Ca^2+^ dynamics in living cells from the perspectives of dynamical systems theory as well as bio-physical and bio-chemical implications in cell biology.

In small biological cells, fluctuations are inherent. Intrinsic fluctuations stemming from random molecular interactions play crucial roles in regulating cellular organizations [28], cellular decision-making, and fate determination [29,30]. Intrinsic fluctuations in intracellular Ca^2+^ oscillations arise due to finite cell size and a small number of reactants [31,32]. One of the present authors [33] has recently studied the stochastic dynamics of intracellular Ca^2+^ oscillations in Houart’s model [24] to investigate the behavior of cytosolic Ca^2+^ dynamics driven by intrinsic fluctuations. In this study [33], a type of entropy known as permutation entropy [34] (based on ordinal patterns in symbolic dynamics) was proposed to characterize and classify diverse dynamical states of the intracellular Ca^2+^ dynamics. The dynamical states considered include steady-state, simple periodic oscillations, bursting, chaos, multiple periodicity, and quasiperiodic oscillations. At the realistic level of intrinsic fluctuation (hereafter referred to as noise) [35–37], the permutation entropy’s ability to distinguish states with multiple-periodicity from chaos remains unsatisfactory. In addition, the permutation entropy analysis was based on representative time series from a limited parameter space of the stochastic Houart’s model, and therefore, the generalizability of its conclusions to larger model parameter space (corresponding to broader dynamical regimes) needs to be further investigated. These limitations raise a fundamental question whether parameter-dependent organizational principles persist under realistic noise levels to remain biologically meaningful and computationally detectable. Answering this question is challenging because the subtle temporal signatures that define these states are often obscured by stochastic fluctuations. One of the most direct ways to address this challenge is to determine whether a robust classifier can successfully distinguish these dynamical states despite the presence of noise. Hence, it is imperative to develop a robust classifier capable of accurately distinguishing dynamical states of intracellular Ca^2+^ concentration with various noise levels.

The classification of dynamical patterns exhibited by intracellular Ca^2+^ concentration holds practical significance from both bio-chemical and bio-physical perspectives. In Ca^2+^ dynamics, a steady state implies a resting state where the intracellular Ca^2+^ concentration is relatively low (~0.1−0.2 μM) [38,39], simple periodic oscillations indicate normal cellular signaling and homeostasis (regulation of accurate control output) [32,40], while bursting is intimately related to apoptotic cell death [41,42]. Quasiperiodicity has been associated with normal functioning and pathology [43,44]. Period-two or period-three bifurcations are linked with multiple cellular phenotypes in yeast [45]. Further, whether chaos is actually present in biological data and how to accurately characterize them still remain open questions of great interest in non-linear physics of complex systems [46–55]. In general, the characterization of diverse dynamical patterns in simulated/real experimental data of complex biological systems, including Ca^2+^ oscillations, is fundamentally significant as these patterns are closely linked with physiological states related to health and disease [56,57].

In this work, we propose using machine learning (ML) to classify the dynamical states of intracellular Ca^2+^ concentration. ML has been widely applied to the analysis of biological processes, including diffusion dynamics in single-particle tracking trajectories [58–60] and gene expression classification from microarray data [61]. The traditional ML approaches, however, often face significant challenges with biological data, which are often noisy, heterogeneous [58], and have limited data points. Further, traditional ML approaches rely on extensive manual feature engineering, which proves to be difficult for such biological data [62]. These difficulties, therefore, hinder ML classification performance. Consistent with this, the experimental data of intracellular Ca^2+^ concentration also face these challenges: experimental traces of Ca^2+^ concentration are not only noisy but also exhibit complex temporal patterns such as bursting, making manual feature extraction highly challenging for accurate classification.

Recent advances in deep learning offer promising solutions to the challenges mentioned above, automatically learning complex temporal patterns directly from raw data, eliminating manual feature engineering requirements [63,64]. A fundamental challenge in applying deep learning to Ca^2+^ dynamics is in obtaining accurate labels for training. This necessitates the use of well-validated synthetic datasets generated from mathematical models to train robust classifiers that can subsequently be applied to experimental data. For this transfer to succeed, the model must effectively generalize dynamical features across different data domains. To achieve this, we employ the large kernel convolutional neural network (LKCNN), which demonstrates superior dynamical feature generalization capabilities for characterization of time series generated by complex systems [65–67]. While conventional deep learning architectures such as residual networks (ResNets) often struggle with the temporal complexity inherent in dynamical systems, LKCNN has demonstrated robust generalization capabilities specifically for complex system characterization, achieving 89.8% accuracy [65]. Unlike standard CNNs with small kernel sizes (~3−5) [68], LKCNN relies on large-scale observational windows, enabling it to capture long-range correlations and complex temporal patterns of various dynamical behaviors. Recent analysis suggests that LKCNN appears well-suited for learning qualitative dynamical features beyond simple periodic patterns, which may explain its superior ability to distinguish between chaotic and regular behaviors across different dynamical systems [66].

In this study, we present a deep learning framework specifically designed for intracellular Ca^2+^ dynamics classification. We extend beyond binary classification approaches used in previous LKCNN studies and distinguish multiple dynamical states: steady states, simple periodic and multiple periodicity oscillations, bursting, chaotic (aperiodic), and quasiperiodic patterns. Although advanced variants such as the Siamese large kernel convolutional neural network (SLKCNN) have been proposed [69], we adopt the original LKCNN architecture as a prototype to investigate the intracellular Ca^2+^ dynamics. Our approach leverages synthetic data from the well-known Houart’s nonlinear model of intracellular Ca^2+^ oscillation in order to train LKCNN classifiers that demonstrate robust generalization to real experimental Ca^2+^ concentration time series data. This methodology enables automated analysis of complex temporal patterns crucial for understanding cellular physiological states and disease mechanisms, eliminating the need for manual feature engineering and expert-dependent pattern recognition.

## Results

### Dynamical patterns of Intracellular Ca^2+^ concentration

**Fig 1:**
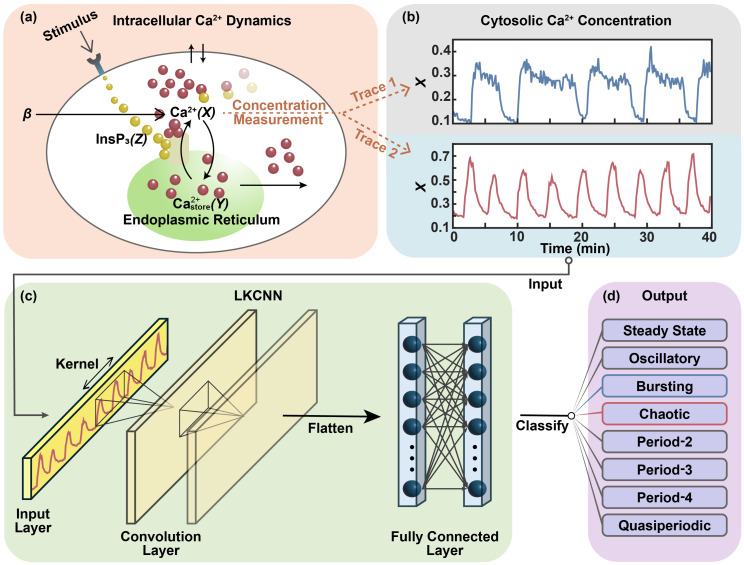
Workflow diagram: Our approach processes diverse dynamical patterns of intracellular calcium (Ca^2^^+^) dynamics in simulated trajectories as well as experimental traces. Simulated data are generated by the interplay between the mechanisms of Ca^2+^-induced Ca^2+^-release (CICR) and Ca^2+^-activated 1,4,5-trisphosphate (InsP_3_) degradation inside a biological cell as illustrated in **(a)**. See Methods section for detailed descriptions of the intracellular Ca^2+^ oscillation model. Experimental traces of Ca^2+^ concentration are obtained from different cells (see the Main text). First, we generate simulated cytosolic Ca^2+^ concentration trajectories from the intracellular Ca^2+^ oscillation model or obtain experimental traces from publicly available datasets (two representative traces shown in **(b)**). We then feed the simulated trajectories or experimental traces to the large kernel convolutional neural network (LKCNN) model (as shown in **(c)**), which classifies the diverse dynamical patterns of cytosolic Ca^2+^ concentration into correct labels **(d)**.

The training of a neural network typically requires large and high-quality datasets with true labels. Since obtaining accurate labels directly from noisy experimental time series data is challenging, we employ a simulation-based approach for training our LKCNN model. One may use a variety of theoretical models that can reliably reproduce the experimentally observed complex temporal patterns of intracellular Ca^2+^ dynamics. In this work, we choose the nonlinear model presented by Houart *et al.* [24] since it can mimic various dynamical states, hence making it suitable for a comprehensive training and classification of intracellular Ca^2+^ dynamics. The nonlinear model centers around the interplay between Ca^2+^-induced Ca^2+^-release (CICR) and Ca^2+^-activated 1,4,5-trisphosphate (InsP_3_) degradation mechanisms, where the coupling of a negative feedback loop (degradation) with a positive CICR cycle gives rise to complex intracellular Ca^2+^ oscillations [26]. A schematic representation of this mechanism is shown in panel (a) of Fig 1, and we detail the governing theoretical nonlinear model in the Methods Section (Eq. (4)).

Chemical kinetics can be described at multiple levels of resolution. At the microscopic level, reactions occur as discrete molecular events that take place at random times. Consequently, the system is characterized by stochastic changes in molecule numbers governed by the chemical master equation. In contrast, when molecule numbers are large, fluctuations become negligible and the dynamics can be described in terms of continuous concentrations evolving deterministically according to rate equations, such as Eq. (4). The chemical Langevin equation (CLE) [70,71] provides an intermediate description: it retains continuous concentration variables while incorporating stochastic terms that account for the intrinsic randomness of reaction events. Within this framework, both InsP_3_ and Ca^2+^ are treated as spatially homogeneous whole-cell concentrations. The relevant molecule counts are therefore evaluated over the full cytoplasmic volume (V≈1−2pL [72,73]). For the physiological concentration ranges considered here (0.1−1μM for InsP_3_ [74,75] and 100−1000nM for Ca^2+^ [76,77]), this corresponds to molecule numbers of order $N\sim 𝒪(10^{4})-𝒪(10^{5})$ for both species, satisfying the large-copy-number condition required for the validity of the CLE [71]. Moreover, under CLE approximation, each reaction contributes both a deterministic drift term and a stochastic noise term arising from counting statistics, whose magnitude scales inversely with the square root of the system volume *V*. As a result, the dynamics approaches deterministic behaviour in large systems but remains strongly fluctuating in smaller systems. This means that the same volume *V* that governs the number of molecules present in the system simultaneously sets the amplitude of stochastic fluctuations around the mean-field trajectory, a dual role that is not a notational coincidence but a direct consequence of converting discrete molecule counts into continuous concentrations within the CLE framework. Moreover, since each reaction event simultaneously alters several species according to the reaction stoichiometry, the same stochastic fluctuation propagates to multiple concentrations. Consequently, a single system-size parameter *V* determines the overall strength of correlated noise throughout the reaction network.

Following this approach, we derive the CLE corresponding to the intracellular Ca^2+^ oscillation model (see the Methods section for a detailed derivation) as follows:


$$\frac{ds}{dt}=[\begin{array}{c} \dot{x}\\ \dot{y}\\ \dot{z} \end{array}]=[\begin{array}{c} V_{0}+V_{1}\beta -V_{2}+V_{3}+k_{f}y-kx\\ V_{2}-V_{3}-k_{f}y\\ \beta V_{4}-V_{5}-\epsilon z \end{array}]$$

$$+\frac{1}{\sqrt{V}}[\begin{array}{c} \sqrt{V_{0}}\xi_{1}+\sqrt{V_{1}\beta }\xi_{2}-\sqrt{V_{2}}\xi_{3}+\sqrt{V_{3}}\xi_{4}+\sqrt{k_{f}y}\xi_{5}-\sqrt{kx}\xi_{6}\\ \sqrt{V_{2}}\xi_{7}-\sqrt{V_{3}}\xi_{8}-\sqrt{k_{f}y}\xi_{9}\\ \sqrt{V_{4}\beta }\xi_{10}-\sqrt{V_{5}}\xi_{11}-\sqrt{\epsilon z}\xi_{12} \end{array}],$$ (1)

where $\xi_{j}$ (j=1,2,…,12) denotes mutually independent Gaussian white noise processes characterized by $\langle \xi_{j}(t)\rangle =0$ and $\langle \xi_{j}(t)\;\xi_{j^{\prime }}(t^{\prime })\rangle =\delta_{jj^{\prime }}\;\delta (t-t^{\prime }).$ While the first term of Eq. (1) is simply the deterministic nonlinear model (Eq. (4)), the second term represents the stochastic component of the intracellular Ca^2+^ dynamics. The prefactor $1/\sqrt{V}$ explicitly accounts for the influence of system size (*V*) or intrinsic fluctuations on the evolution of Ca^2+^ concentration Specifically, the parameter *V* determines the amplitude of intrinsic fluctuations: as *V* increases, the relative contribution of stochastic effects diminishes. In the thermodynamic limit, where V→∞, the term $1/\sqrt{V}$ tends to zero, causing intrinsic fluctuations to vanish altogether. These intrinsic fluctuations manifest as inherent noise in the underlying dynamics, making classification difficult. To clarify how system size *V* is related to intrinsic fluctuation in the dynamics, we refer the readers to the derivation of Chemical Langevin formalism presented in Methods Section.

**Fig 2:**
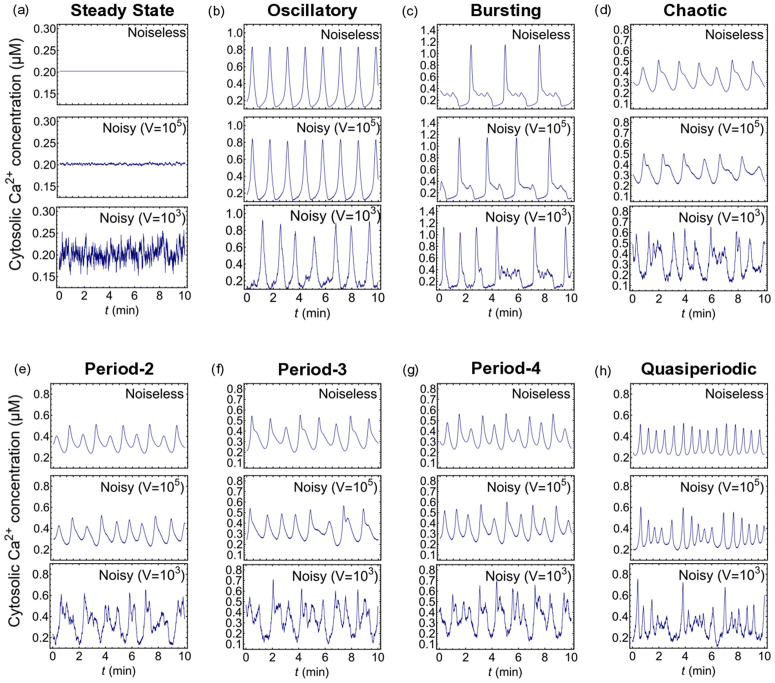
Various dynamical states of cytosolic Ca^2^^+^ concentration: (a) Steady state, (b) oscillating state, (c) bursting, (d) chaotic state, sequences of (e) period-2, (f) period-3, (g) period-4, and (h) quasiperiodic oscillations. For each panel, the three rows correspond to deterministic or noiseless (*top*), noisy with *V* = 10^5^ (*middle*), and *V* = 10^3^ (*bottom*).

Numerically solving the CLE (1) using the Euler-Maruyama method for different values of the rate constants and other model parameters (detailed in the Methods Section), concentrations *x*, *y*, and *z* in Eq. (1) exhibit various kinds of dynamics. For visual reference, representative trajectories are presented in Fig 2, which illustrates characteristic temporal patterns in cytosolic Ca^2+^ concentration *x*(*t*) (expressed in μM) at three levels of noise $V=\infty ,10^{5}$ and 10^3^. The panels illustrate: (a) steady state, (b) simple periodic oscillations (oscillatory), (c) bursting, (d) chaotic, (e) period-2 (P-2), (f) period-3 (P-3), (g) period-4 (P-4), and (h) quasiperiodic oscillations. Time *t* is measured in minutes (min).

We use the dynamical states of cytosolic Ca^2+^ concentration *x*(*t*) as inputs for our LKCNN classification (as illustrated in panels (b) and (c) of Fig 1). Physiological bounds on cytosolic Ca^2+^ concentration, typically from below 0.1μM up to 1μM with oscillation periods ranging from a few seconds to ~30 min [24,78], limit the parameter space of interest. The model in Eq. (1) however contains 19 tunable parameters whose variation can generate a wide range of dynamical states. For training and testing our neural network, we therefore consider three parameter regions that together largely cover the observed amplitude and frequency range of Ca^2+^ oscillations. These regions have been numerically analyzed previously in the original study [24], and we provide the exact values of the parameters used in Table 1 provided in the Methods section. However, in our study, we further subdivide the parametric region close to chaos into multiple-periodicity states, namely period-2 (P-2), period-3 (P-3), and period-4 (P-4) oscillations to better highlight the advantages of the deep-learning approach.

**Table 1:** Parameter values used in the numerical simulation of the intracellular calcium oscillation model (1). Each region exhibits distinct dynamical behaviors: Region 1 (Bursting, Oscillatory, Steady State), Region 2 (Chaotic, Period-2, Period-3, Period-4, Steady State, Oscillatory), and Region 3 (Quasiperiodic, Steady State, Oscillatory). Within each region, 1000 synthetic trajectories were generated per dynamical pattern, each for training and testing the LKCNN classifier.

Parameters	Region 1	Region 2	Region 3
β	[0.0, 0.8]	[0.65, 0.8]	[0.0, 0.8]
$\epsilon \;({min}^{-1})$	[0.0, 10.0]	[10.0, 16.8]	[0.0, 2.5]
$V_{0}\;(\mu M\;{min}^{-1})$	2	2	2
$V_{1}\;(\mu M\;{min}^{-1})$	2	2	2
$V_{M2}\;(\mu M\;{min}^{-1})$	6	6	6
$k_{2}\;(\mu M)$	0.1	0.1	0.1
$V_{M3}\;(\mu M\;{min}^{-1})$	20	30	20
$k_{x}\;(\mu M)$	0.3	0.6	0.5
$k_{y}\;(\mu M)$	0.2	0.3	0.2
$k_{z}\;(\mu M)$	0.1	0.4	0.2
$V_{M5}\;(\mu M\;{min}^{-1})$	30	50	30
$k_{s}\;(\mu M)$	0.6	0.3194	0.3
$k_{d}\;(\mu M)$	1	1	0.5
$k_{f}\;({min}^{-1})$	10	1	1
$k\;({min}^{-1})$	10	10	10
$V_{4}\;(\mu M\;{min}^{-1})$	3	2	5
*m*	4	2	2
*p*	1	1	2
*n*	2	4	4

### Optimization of LKCNN architecture

We employ the LKCNN architecture shown in panel (c) of Fig 1 to classify the various dynamical states of intracellular Ca^2+^ dynamics at varying levels of noise (quantified by *V*), leveraging its demonstrated effectiveness in dynamical systems analysis [65,66]. However, prior to training and classifying the dynamical states, we have to properly initialize the neural network. The initialization is set through the seed, which determines the weights of the LKCNN before training. Our analyses indicate that the choice of seed has only a minimal impact on the overall classification performance, highlighting the robustness of the LKCNN classifier. The other parameter is the kernel size *k*, which is defined as the network’s temporal receptive field. It is the span of consecutive data points of a time series that it analyzes simultaneously to detect patterns. This value influences how much local context the neural network considers when extracting features from the input data. Previous studies have demonstrated the effectiveness of LKCNN by using a fixed kernel size of 100 for the binary classification of chaotic versus regular dynamics in noise-free systems [65,66], showing that large kernels enable the direct capture of extended temporal dependencies and long-range correlations that characterize complex dynamical behaviors. For instance, long-range patterns like P-3, P-4, and bursting necessitate a large temporal receptive field for effective detection. However, a trade-off exists: an excessively large kernel, while beneficial for capturing these long-term dependencies, can obscure the fine-grained details of shorter-term patterns by blurring their local features. Since the amplitude, frequency, and noise levels of the Ca^2+^ concentration dynamics all lie within well-defined, experimentally-determined ranges, the kernel size used for classifying the dynamical patterns is also expected to perform optimally only within a specific range. Therefore, identifying an optimal kernel size that is large enough for long-range correlations yet fine enough to preserve local details is crucial for accurately distinguishing the full spectrum of Ca^2+^ dynamical behaviors. Operationally, the LKCNN architecture proceeds in such a way that the large kernel slides across the input Ca^2+^ concentration time series to extract temporal features through successive convolutional layers and the extracted features are then flattened and processed through fully connected layers to produce the final classification output (as illustrated in Fig 1(d)). The detailed LKCNN architecture and training procedure are described in the Methods section.

We now analyze the performance of the LKCNN classifier across a range of kernel sizes *k* under two distinct scenarios of cytosolic Ca^2+^ concentration: (i) an ideal, noiseless condition, and (ii) a more realistic, noisy condition. This allows us to investigate the impact of noise on the classifier’s performance as well as to identify a range of kernel sizes that demonstrate robustness in noisy conditions. In the first setup, which we refer to as the “Noiseless” scenario, we evaluate the performance of the classifier on the noise-free (V=∞, deterministic dynamics) test data. The second setup referred to as “Noisy” scenario assesses our classifier’s robustness against test data with a wide range of *V* values necessarily including the realistic noise levels ($V\in \{10^{6},10^{5}\}$) [33,35–37] of intracellular Ca^2+^ dynamics. For both setups, we train the LKCNN on a dataset spanning noise levels $V\in \{\infty ,10^{8},10^{7},10^{6},10^{5}\}$. For each value of *V*, the training dataset is created using at least 1,000 trajectories from each of the eight dynamical states of Ca^2+^ dynamics. Similarly, another dataset with 12,000 trajectories was generated to test the performance of the classifier. Since our synthetic datasets contain time trajectories of various dynamical states (hereafter also referred to as labels or class) of cytosolic Ca^2+^ concentration, we quantify our classifier’s performance as its ability to solve a multi-class classification problem, hence termed classification accuracy. That is, the machine learning task is to predict and correctly assign one of the eight distinct dynamical pattern labels to each test data sample. We define the classification accuracy as the fraction of samples for which the LKCNN classifier’s predicted label exactly matches the true label as:


$$\text{Classification Accuracy}=\frac{\sum_{i}N_{i}^{C}}{\sum_{i}N_{i}^{C}+\sum_{i}N_{i}^{M}},$$ (2)

where $N_{i}^{C}$ and $N_{i}^{M}$ represent respectively the number of correctly classified and misclassified samples *i* (steady state, bursting, oscillatory, P-2, P-3, P-4, chaotic, and quasiperiodic oscillations).

**Fig 3:**
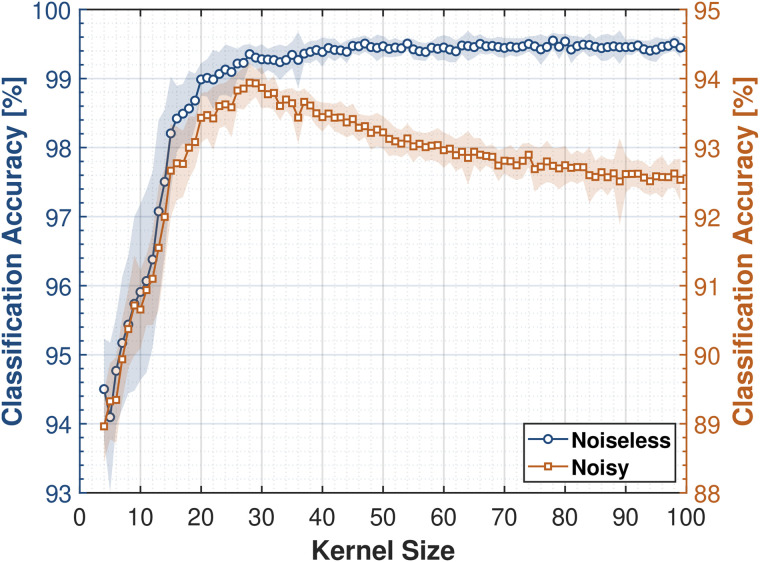
Classification accuracy of LKCNNs over a range of kernel size for noiseless (blue circles) and noisy (orange squares) trajectories of cytosolic Ca^2^^+^ dynamics. For noisy trajectories, we train the LKCNN on data with noise levels $V\in \{\infty ,10^{8},10^{7},10^{6},10^{5}\}$ and test on data with $V\in \{10^{6},10^{5}\}$. The solid lines indicate the mean accuracy averaged over a set of 20 distinct randomly generated seeds for each kernel size. The shaded envelopes denote one standard deviation (±σ) calculated from the multiple realizations.

To ensure reproducibility and determine the optimal architecture, we train and subsequently test the accuracy of the LKCNN classifier with various seeds and kernel sizes. Fig 3 presents the classification accuracy for the noiseless (blue circles) and noisy (orange squares) test datasets against the kernel size *k* of the LKCNN (we use k=4,5,6,…,100). We take the same set of 20 distinct randomly generated seeds for each k value, ensuring that performance differences across k are not attributable to different random initializations. The solid lines indicate the mean accuracy averaged over the multiple seeds. The shaded envelopes denote one standard deviation (±σ), indicating the stability of our LKCNN classifier’s performance. We observe a narrower band of blue shaded envelope under noiseless conditions as compared to the noisy condition at k≥30, implying a more stable performance for the noise-free or deterministic dynamics of cytosolic Ca^2+^ concentration across different random seeds. We further observe that the presence of noise significantly lowers the multi-class classification performance of the classifier, indicated by the lower accuracy values of the noisy data condition compared to the noiseless case. Notably, we observe that the two cases show distinct relationships between the LKCNN kernel size and classification accuracy. In the noiseless scenario, the accuracy (blue curve) rapidly improves with an increase in kernel size, followed by a saturation around k~30. On the other hand, for the noisy data condition, a more realistic scenario, the accuracy curve (orange) shows a clear peak for kernel sizes ~25−30, indicating an optimal classification performance of the LKCNN within this range. Beyond this range, the performance of the classifier steadily declines, which indicates that while larger kernels are beneficial for the noiseless data condition, moderately-sized LKCNN kernels are optimal for datasets with realistic noise levels. We thus obtain an optimal LKCNN architecture for robust classification of the intracellular Ca^2+^ dynamics from synthetic numerically-simulated trajectories that can be used for analysis of real experimental Ca^2+^ concentration traces. For all subsequent analyses, we use a kernel size of *k* = 28 because it yields the best performance in both noisy and noise-free settings.

### Classification of noiseless Ca^2+^ states

Having obtained the optimal LKCNN classifier, we now analyze its performance at the level of individual dynamical states. Rather than relying solely on aggregate accuracy, we report per-state accuracy, precision, recall, and F1 scores to reveal any heterogeneity across the eight states. For each class *i* (one-versus-rest), let $N_{i}^{C}$ (true positives) and $N_{i}^{M}$ (false negatives) denote the numbers of correctly classified and misclassified trajectories with true label *i*, respectively, consistent with the notation used in the accuracy definition. We further let $N_{i}^{FP}$ denote the number of trajectories whose true label is not *i* but are predicted as class *i* (false positives). The precision, recall, and F1 score for class *i* are then given by


$${Precision}_{i}=\frac{N_{i}^{C}}{N_{i}^{C}+N_{i}^{FP}},$$

$${Recall}_{i}=\frac{N_{i}^{C}}{N_{i}^{C}+N_{i}^{M}},$$

$${F1}_{i}=\frac{2\;{Precision}_{i}\;{Recall}_{i}}{{Precision}_{i}+{Recall}_{i}}.$$ (3)

We further examine the confusion matrix to identify systematic misclassifications and shared error modes between closely related patterns.

We focus on the noiseless case in this section. We continue with the optimized LKCNN classifier trained on the noise levels of $V\in \{\infty ,10^{8},10^{7},10^{6},10^{5}\}$ with *k* = 28, and subsequently evaluate its performance on a distinct test set of 12,000 noiseless trajectories. Table 2 reports class-wise performance, indicating a uniformly high accuracy of 99.4%. We find that precision, recall, and F1 are near unity for most states. Steady state, bursting, oscillatory, and quasiperiodic are effectively identified. The multiple-periodicity states P-2, P-3 and P-4 are discerned with high precision but slightly lower recall (~0.969−0.996), yielding F1 scores of 0.985 (P-2), 0.982 (P-3), and 0.981 (P-4). Performance is comparatively weaker for chaotic, suggesting a tendency toward false positives in this class. Overall, the LKCNN works effectively as a classifier for all states, with minor errors concentrated in chaotic and, to a lesser extent, P-3 and P-4 states. The numerical values of these metrics for the eight dynamical states are given in Table 2.

**Table 2:** Precision, recall, and F1 score for noiseless simulated trajectories of cytosolic Ca^2+^ concentration.

Dynamical Pattern	Precision	Recall	F1 score
Steady State	1.0000	1.0000	1.0000
Bursting	0.9999	1.0000	0.9999
Oscillatory	0.9999	0.9996	0.9998
Period-2	0.9756	0.9957	0.9855
Period-3	0.9900	0.9733	0.9815
Period-4	0.9930	0.9691	0.9809
Chaotic	0.9643	0.9867	0.9753
Quasiperiodic	1.0000	0.9999	0.9999

**Fig 4:**
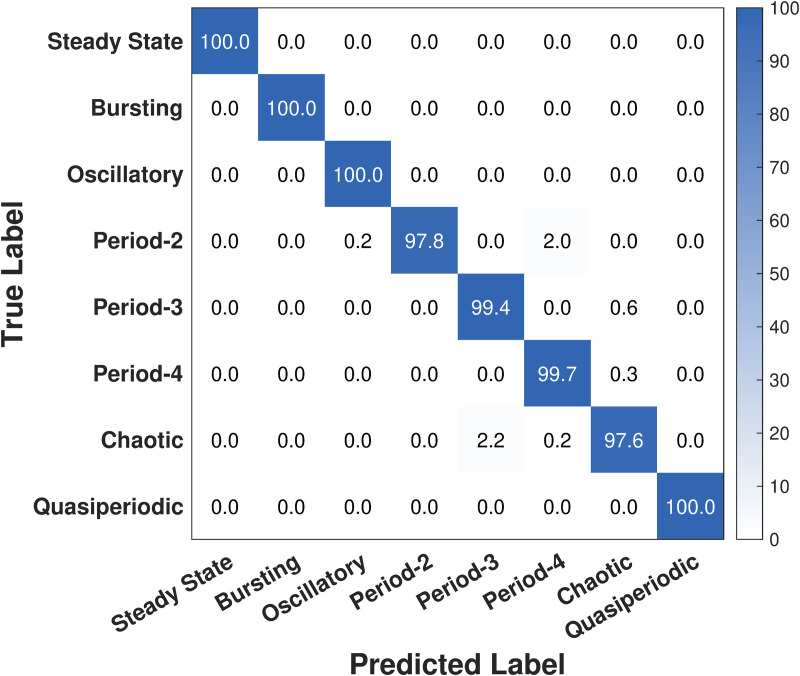
Confusion matrix showing the result of multi-class classification performance of our LKCNN model for the noiseless case. Diagonal entries denote the percentage of correctly predicted dynamical states of cytosolic Ca^2+^. Labels indicate steady state, bursting, oscillatory, period-2, period-3, period-4, chaotic state, and quasiperiodic oscillation.

We continue our analysis with the confusion matrix shown in Fig 4 in which the rows correspond to the true labels of the simulated cytosolic Ca^2+^ concentration trajectory data, while the columns represent the labels predicted by our LKCNN classifier. The diagonal entries (blue colored) show the percentage of each class that has been correctly identified (per-class accuracy), whereas off-diagonal values indicate misclassifications. Our result shows that the optimized LKCNN achieves a high level of classification performance on the noiseless test data, achieving an overall average accuracy of 99.5%. The diagonal values, many of which are nearly 100.0%, thus indicate near-perfect classification for most dynamical states of cytosolic Ca^2+^ concentration, including steady state, bursting, oscillatory, P-3, P-4, and quasiperiodic oscillations. We see that a small fraction of P-2 samples are confused with P-4 (2.0%), and a small fraction of chaotic trajectory samples are misidentified, primarily as P-3 (2.2%). These misclassifications suggest that while our LKCNN classifier is highly effective, its most significant challenge lies in distinguishing between the fine-grained features that separate chaotic behavior from certain multiple-periodicity states. Moreover, the asymmetric P-2 → P-4 confusion is concentrated in trajectories with larger cycle-to-cycle amplitude variability (see S1 Fig). Nonetheless, the high classification accuracy shown on the diagonal of the confusion matrix demonstrates the excellent distinguishing power and reliability of our LKCNN classifier in noise-free datasets.

### Classification of noisy Ca^2+^ states

We now extend our analysis to examine the performance of the optimized LKCNN classifier at the level of individual dynamical states in the presence of noise. For a more comprehensive analysis of the noise effect, we extend both the training and test datasets to cover increasingly noisy conditions, corresponding to smaller *V* values. While the kernel size *k* is fixed at 28 as stated earlier, the present configuration is fundamentally different because training is performed across a wider range of noise levels, $V\in \{\infty ,10^{8},10^{7},10^{6},10^{5},10^{4},10^{3}\}$. A broader and more extreme spectrum of noisy conditions is considered to improve the robustness of the LKCNN classifier and better capture the dynamical behavior of Ca^2+^ states under challenging test scenarios. This setup also allows us to investigate performance over more distinct noise levels than before.

**Fig 5:**
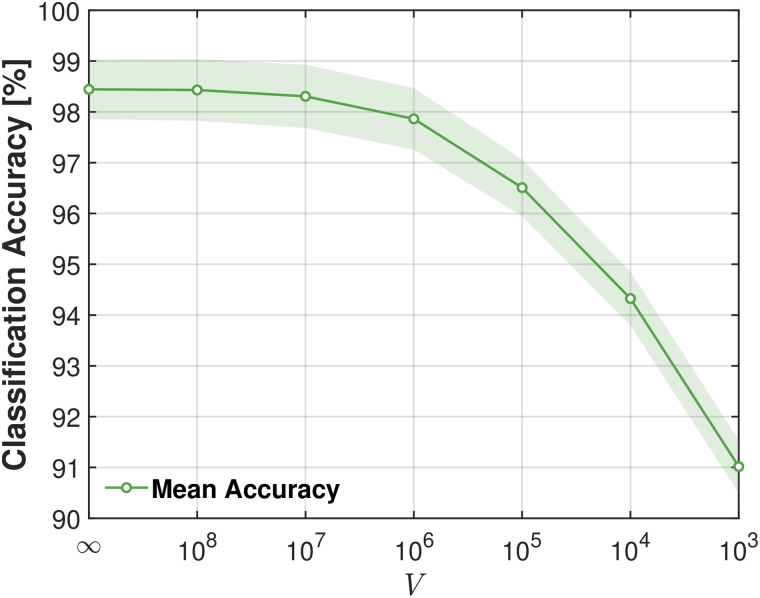
Classification accuracy of LKCNN versus system size *V* for the simulated cytosolic Ca^2^^+^ dynamics. Noise in the trajectories scales inversely as the system size as $\sim 1/\sqrt{V}$, i.e., the smaller the system size *V*, the larger the noise in the trajectory data. The green circles denote the mean accuracy averaged over a set of 20 distinct randomly generated seeds for each noise level. The shaded green region indicates the corresponding standard deviation (±σ) from multiple realizations.

Fig 5 shows how the classification accuracy of the LKCNN classifier varies with the cumulative noise level in the test dataset. Here, “cumulative” means that the noise level *V*, plotted on the *x*-axis, represents the maximum noise level included in the test set. For example, *V* = 10^6^ indicates that the test set contains all samples from $V\in \{\infty ,10^{8},10^{7}$, 10^6^}. Similarly, the accuracy value at *V* = 10^3^ is computed using the entire test dataset from V=∞ through *V* = 10^3^. The cumulative test set mimics a realistic condition by progressively broadening the distribution of noise in the evaluation data, as in experimental scenarios, a deployed classifier would not see only one fixed noise level, and would face a mix of conditions. The green circles in Fig 5 denote the mean accuracy over 20 runs with different random seeds for each cumulative noise level, while the shaded green region indicates the corresponding standard deviation (±σ). The highest accuracy (≈98.5%) occurs when testing on the noiseless dataset (V=∞). As noise is progressively added, mean accuracy steadily declines, remaining relatively high (>rsim98.0%) for $V\geq 10^{6}$, but dropping more noticeably when $V\leq 10^{5}$, reaching ≈91.0% at *V* = 10^3^. These results show that the quality of the input data inherently constrains the LKCNN’s classification performance. Note that for V=∞, the accuracy of 98.5% is slightly below the > 99.0% achieved in Fig 3, as the training set for the latter was limited to $V\in \{\infty ,10^{8},10^{7},10^{6},10^{5}\}$. This suggests that including extremely noisy data (*V* = 10^3^) in training slightly degrades performance even when testing on noiseless data. Indeed, when training is restricted to noise levels up to *V* = 10^4^ (excluding *V* = 10^3^), performance on the noiseless test set almost matches the results in Fig 3.

Restricting our subsequent analysis to realistic noise levels in Ca^2+^ dynamics ($V\in \{10^{6},10^{5}\}$ [33]) at which the classification accuracy remains above 96%, we ensure the evaluation reflects physiologically relevant conditions. We use the same optimal LKCNN with kernel size *k* = 28 as before. We construct the training dataset by combining 12,000 samples from each of the *V* levels ($\left\{\infty ,10^{8},10^{7},10^{6},10^{5}\right\}$), resulting in a total of 60,000 training samples. The test set is then constructed with the exact same distribution as the training data, also containing 12,000 samples from each of the five *V* levels for a total of 60,000 test samples. Table 3 summarizes the class-wise performance on the noisy set, showing high but non-uniform accuracy (overall = 94.00%). Bursting is essentially perfectly identified, and steady state, oscillatory, and quasiperiodic also perform good (F1 scores >rsim0.980). Performance is lower for the multi-periodicity states P-2, P-3, and P-4 (F1 scores ≈0.840−0.860), with P-2 mainly limited by precision, and both P-3, P-4 mainly limited by recall. The weakest class is chaotic (F1 ≈0.781), indicating substantial confusion with other labels. Overall, LKCNN remains robust under noise for most behaviors, with errors concentrated in chaotic and, to a lesser extent, the multi-periodicity states. Moreover, on comparing with the metrics for the noiseless data, we note a clear reduction in the overall accuracy of approximately 5.41%. An examination of the F1 scores across individual classes reveals that the most substantial performance degradation occurs in the chaotic class. Other classes exhibiting notable decreases include P-2, P-3, and P-4. These findings suggest that such classes are particularly sensitive to the introduction of noise, possibly due to the perturbation of key discriminative features within their representations. In contrast, several classes demonstrate substantial robustness to noise. The steady state, for example, experiences only a marginal reduction in F1 score from 1.000 to 0.996. Similarly, the bursting and quasiperiodic classes maintain relatively stable performance under noisy conditions, indicating that their underlying feature patterns remain discernible despite the fluctuations.

**Table 3:** Precision, recall, and F1 score for noisy simulated trajectories of cytosolic Ca^2+^ concentration.

Dynamical Pattern	Precision	Recall	F1 score
Steady State	0.9939	0.9982	0.9960
Bursting	1.0000	0.9980	0.9990
Oscillatory	0.9864	0.9893	0.9878
Period-2	0.8379	0.8922	0.8637
Period-3	0.8674	0.8190	0.8420
Period-4	0.8608	0.8234	0.8415
Chaotic	0.7665	0.7971	0.7811
Quasiperiodic	0.9993	0.9898	0.9945

**Fig 6:**
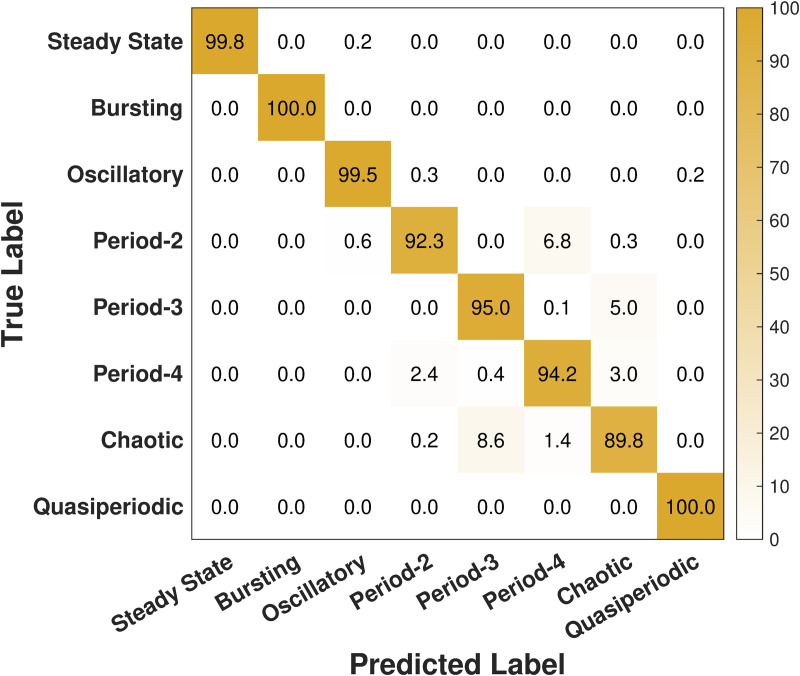
Confusion matrix showing the result of multi-class classification performance of our LKCNN model for the case of realistic noise levels. Diagonal entries denote the percentage of correctly predicted dynamical states of cytosolic Ca^2+^. Labels indicate steady state, bursting, oscillatory, period-2, period-3, period-4, chaotic state, and quasiperiodic oscillation.

Fig 6 presents the confusion matrix for test datasets containing realistic noise levels of intracellular Ca^2+^ dynamics, where we find an overall average accuracy to be 97.42%. While this value is still a high accuracy, it represents a noticeable drop from the previous 99.54% achieved in the noiseless case (Fig 4). A detailed breakdown of the confusion matrix of Fig 6 shows how this performance degradation is distributed across the different dynamical classes. We see that the diagonal entries (orange colored) of some of the classes are lower than those observed for the noiseless case (Fig 4), indicating an increase in misclassifications for these classes. We state some key findings from Fig 6 as follows. The confusion among different multi-periodicity states has worsened from before. For example, 6.8% of P-2 samples are incorrectly labeled as P-4. The confusion between chaos and periodic states (P-2, P-3, and P-4) has noticeably increased. A significant 8.6% of true chaotic samples are now misclassified as P-3. Conversely, 5.0% of P-3 samples and 3.0% of P-4 samples are misidentified as chaotic. These results demonstrate that the high accuracy observed in the case of noiseless datasets is significantly affected when noise is present, particularly between chaotic and multiple-periodicity states.

### Generalization capability to experimental data

Having trained and validated the LKCNN classifier exclusively on synthetic datasets, we now turn to its deployment for practical use. In order to bridge the gap between model development and real-world applicability, we use the optimized and tested classifier to process experimental measurements as input and assign them to the appropriate classes. While the network has been exposed only to controlled synthetic data during training, deploying it on experimental data allows us to evaluate its generalization capacity and robustness under realistic conditions.

To evaluate the generalizability of our optimized LKCNN classifier, we now construct a dataset of Ca^2+^ concentration experimental data from two sources: (a) pancreatic islets of mouse β-cells [79,80] displaying diverse patterns in Ca^2+^ concentration recorded in eight genetically distinct mouse strains, and (b) WT-HEK293, STIM-KO, and ORAI TKO cells [81,82] exhibiting a diversity of Ca^2+^ traces upon different agonist stimulation conditions, like varying concentrations of carbachol (CCh). In the absence of any physiologically motivated ground-truth classification, we rely on manual labeling of experimental traces wherever possible. From the available dataset, we conduct a careful visual screening of the time series to identify clearly distinguishable dynamical regimes. While the dataset comprises a large number of time series, only those with unambiguous dynamical features are deemed suitable for classification. The characteristic features of bursting dynamics include a plateau fraction (time spent in the active oscillation phase) followed by a silent duration phase in the temporal pattern. Steady states are characterized by relatively low (~0.1−0.2μM) concentration. However, it proves difficult to visually identify all other states due to the noise levels as well as the length of data available in the experimental data sets. Following this criterion, we identify 46 time series in the bursting class and 39 time series in the steady state class, and 41 are manually classified as “others” and include visually indistinguishable classes like chaos, oscillatory, quasi-periodic and the multiple-periodicity states. Therefore, the curation process yields a total of 126 in three classes, namely (i) steady state, (ii) bursting, and (iii) others. Since the dataset contains only Fura-2 ratio measurements of intracellular Ca^2+^ concentration, we have employed the Grynkiewicz equation [83] with estimated calibration parameters to convert the ratio data into approximate Ca^2+^ concentrations. Moreover, we have used linear interpolation to make the experimental traces of exactly 1000 data points, making it suitable for the optimized LKCNN. Using this procedure, we create a benchmarking dataset for validating the performance of our LKCNN classifier on experimental Ca^2+^ concentration dynamics.

**Fig 7:**
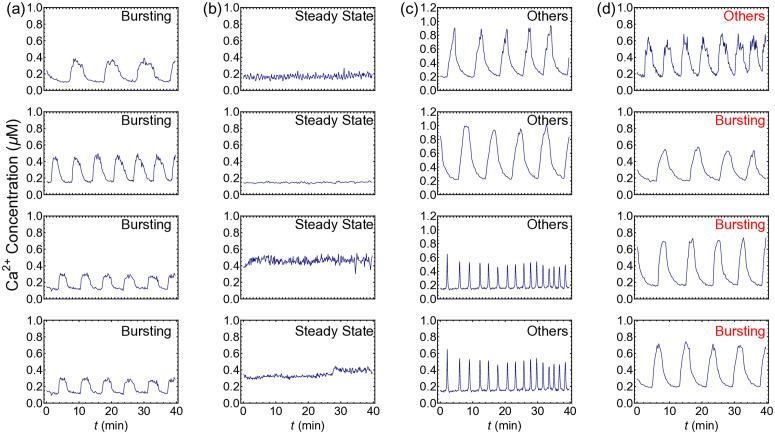
A sample of experimental trajectories with the LKCNN predicted class. Correctly classified **(a)** Bursting, **(b)** Steady States, **(c)** Oscillatory states. **(d)** Misclassified states where the first state was manually labelled as Bursting, while the other three were labelled as Others.

**Fig 8:**
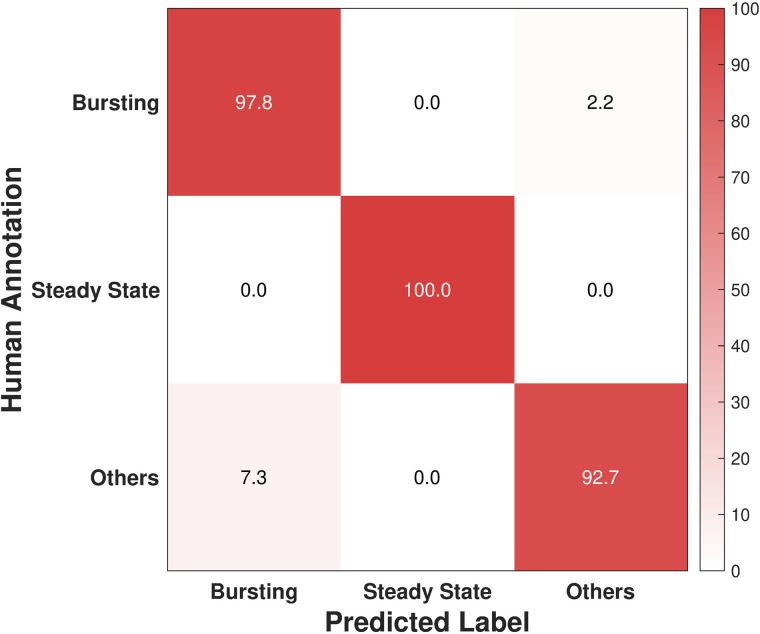
Confusion matrix showing the classification performance of our LKCNN model for the experimental dataset. Diagonal entries denote the percentage of correctly predicted dynamical states based on human annotation. Labels indicate three representative classes: Bursting, Steady State, and Others.

When applied to the experimental dataset, the LKCNN has successfully produced stable classifications across all inputs. Representative examples of the experimental trajectories, together with their correctly classified labels, are shown in panels (a), (b), and (c) of Fig 7. Out of the entire set of 126 time series, only one bursting trajectory and three trajectories marked as others were misclassified, resulting in an overall accuracy of 96.8%. These four misclassified trajectories are shown in panel (d) of Fig 7 with their LKCNN-predicted labels indicated by the text in red color. While the trajectory in the upper panel is manually labeled as bursting, LKCNN predicts it as a chaotic state. The other three trajectories were manually classified as others, but predicted as bursting by the LKCNN classifier. The accuracy percentages of each class are provided in the confusion matrix in Fig 8. The full classification results for all 126 time series, along with the curated data and the analysis scripts, are available in our GitHub repository. Note that the accuracy found in this section does not reflect the true performance of the LKCNN classifier, as the test dataset is highly curated. While the validation dataset may be subject to human errors, the true advantage of the LKCNN lies in its ability to operate beyond visually distinguishable data, providing fast and reliable classification. The results in the section provide an essential proof of concept, demonstrating not only how the model can be integrated into a data analysis pipeline but also how well it performs when confronted with the variability and noise inherent in experimental observations.

### Comparison with conventional machine learning classifiers

We compare the performance of the LKCNN model against two widely adopted conventional machine learning classifiers: the Support Vector Machine (SVM) with a linear kernel [84] and the Random Forest (RF) [85]. Both methods serve as natural baselines given their broad applicability and well-established use in biological time series classification [86]. To ensure a fair and unbiased comparison, both classifiers were trained on Fast Fourier Transform (FFT) features extracted from the identical training set used for the LKCNN and evaluated on the same test sets. FFT-based features are a standard and effective representation for oscillatory time series, as they encode the dominant frequency content and harmonic structure of each trajectory into a fixed-length feature vector suitable for conventional classifiers [86].

**Fig 9:**
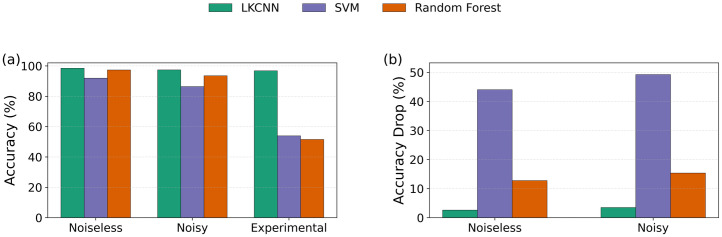
Comparison of LKCNN against conventional baselines and robustness to spike-like corruption. **(a)** Classification accuracy (%) of LKCNN versus two conventional classifiers, namely, linear-kernel SVM and Random Forest (see legend at the top of the figure) on noiseless synthetic trajectories, noisy synthetic trajectories, and experimental Ca^2+^ traces. SVM and RF operate on FFT-based features of the trajectories. **(b)** Accuracy drop (%) after adding sparse, large-amplitude impulsive noise to the synthetic test trajectories. Accuracy drop is computed relative to the corresponding uncorrupted test set.

The classification results are shown in Fig 9(a). Under noiseless conditions, both SVM and RF achieve reasonable accuracy, with RF (97.4%) marginally outperforming SVM (92.0%). However, both classifiers exhibit a pronounced degradation in performance as noise is introduced, with SVM declining to 86.4% and RF to 93.5% under noisy conditions. When applied to experimental Ca^2+^ data, the degradation is substantially more severe: SVM drops to 54.0% and RF to 51.6%, representing poor discriminative performance. In contrast, the LKCNN retains strong generalisation across all conditions, demonstrating a markedly smaller performance gap between synthetic and experimental data. Per-class performance metrics for SVM and RF are provided in S2 Fig.

To further probe the robustness of each classifier to signal corruption unrelated to intrinsic Ca^2+^ dynamics, we subject the synthetic test sets to impulsive noise — a form of distortion characterised by large-amplitude spikes at randomly selected time points, which is representative of artefacts commonly encountered in fluorescence imaging data [87]. Representative corrupted time series are shown in S3 Fig. The accuracy drop (%) under impulsive noise is reported in Fig 9(b). RF exhibits a substantial accuracy drop of 12.8% and 15.4% under noiseless and noisy conditions, respectively, while SVM shows an even larger degradation of 44.1% and 49.3%. In contrast, the LKCNN shows a markedly smaller drop of only 2.6% and 3.5% under the same conditions.

This robustness can be attributed to the large temporal receptive field of the LKCNN, which captures long-range dynamical structure spanning multiple oscillation cycles. Because the learned representation integrates information over many time steps, sparse outlier spikes contribute only marginally to the overall feature extraction, leaving the classification largely unaffected. In contrast, FFT-based features are sensitive to large-amplitude artifacts because a single corrupted time point perturbs the global spectral representation of the entire trajectory, disproportionately degrading classifier performance.

## Discussion

Biological rhythms are central to life [47]. Some examples of biological oscillations include circadian rhythms, ovarian cycles, hormonal rhythms, mitotic cycles, and calcium oscillations [88]. Long-standing questions as to how we can decode the fluctuations in these physiological rhythms for meaningful information (for instance, patterns of the rhythms or anomalies in rhythmic time periods) that can help in a deeper understanding and better diagnosis of human disease remain active areas of research. Deep learning techniques combined with biomedical research can transform our understanding of the rhythms of life. Our present work is motivated by this research direction and addresses a fundamental question about whether dynamical organizational principles persist under realistic biological noise levels. The successful performance of our LKCNN classifier in distinguishing diverse dynamical patterns of intracellular Ca^2+^ concentration through realistic biological noise provides evidence that these organizational signatures are robustly preserved to a substantial extent. This suggests that the dynamical patterns underlying calcium signaling can be detectable and biologically meaningful even under noisy conditions, effectively preserving information about the cell’s underlying dynamical state.

This conclusion is supported by several key achievements of our LKCNN framework. We classified a broad range of intracellular Ca^2+^ dynamical regimes with high accuracy, extending the task from binary classification to an eight-class problem that captures the diversity of dynamical behaviors underlying calcium signaling. Systematic evaluation of kernel size reveals that intermediate temporal receptive fields are optimal for balancing sensitivity to short-lived fluctuations with the ability to recognize longer-range temporal dependencies. These findings indicate the architectural flexibility of LKCNNs in extracting features from complex biological time series and provide insights into the temporal scales most relevant for dynamical pattern recognition in cellular systems.

Our analysis also highlights the impact of intrinsic stochasticity on classification accuracy. Whereas steady state, bursting, and simple oscillatory regimes were robustly distinguished, multiple-periodicity and chaotic dynamics were more difficult to resolve under elevated noise. This difficulty reflects a genuine overlap in their temporal signatures, making a clear separation a fundamental challenge. Indeed, a previous study using statistical measures confirmed this challenge, demonstrating that the distinction between these states diminishes under large intrinsic fluctuations [33]. While such measures are powerful for characterizing the complexity of known states, they often lack the clear decision boundaries required for robust classification. In contrast, our deep-learning approach is specifically designed for this classification task. Remarkably, it achieves an overall accuracy of over 90% even when the testing data set contained trajectories with noise levels two orders larger than its realistic value. This demonstrates that for the practical goal of automating the labeling of complex cellular dynamics, the feature-learning capabilities of the LKCNN framework offer a significant and robust advancement over traditional statistical methods.

Importantly, the application of LKCNN to experimental Ca^2+^ concentration traces demonstrated strong agreement with expert annotations, confirming its generalizability beyond synthetic datasets. On the same experimental dataset, conventional baselines (SVM and RF) exhibited markedly degraded performance and pronounced vulnerability to impulsive noise artifacts. This validation underscores the potential of LKCNNs as automated tools for high-throughput and unbiased classification of cellular dynamics. More broadly, the framework is well-positioned to extend to other oscillatory processes, including neuronal firing [89,90], circadian rhythms [91,92], and transcriptional dynamics [93]. Together, these results demonstrate the value of LKCNNs in bridging computational modeling and experimental biology, enabling scalable and systematic investigation of complex cellular behaviors.

Although the optimized LKCNN classifier demonstrated strong performance, this study is subject to three key limitations. First, the analysis was conducted assuming that the system remains within a single dynamical regime. However, in experimental contexts, external perturbations such as nutrient fluctuations can induce transitions across multiple regimes, a complexity not captured by the present framework. Second, the influence of intrinsic fluctuations on system stability, particularly near phase boundaries where birhythmic systems may stochastically transition between states, was largely excluded from consideration, despite its potential importance for classifier robustness. Third, the reliance on manually annotated experimental data introduces the possibility of bias and inconsistency, whereas labels derived from physiological markers would offer a more objective and reproducible foundation. Addressing these limitations presents promising avenues for future work, which could further enhance the reliability and applicability of the proposed framework.

Beyond addressing these technical limitations, another significant avenue for future research lies in extracting deeper biological insights. Convolutional neural networks with various kernel sizes have demonstrated a strong ability to uncover biologically meaningful patterns in complex biological systems, including circRNA–RBP interactions [94], Alzheimer’s disease neuroimaging analysis [95], neural interaction decoding in brain activity [96], and selection inference in genomic data [97,98]. Similarly, we can extend our work to provide biological insight by leveraging LKCNN-based classification to constrain the bifurcation landscape governing Ca^2+^ dynamics. The dynamical regimes identified by the LKCNN do not exist as independent categories but rather as distinct regions of the underlying bifurcation landscape, parameterised principally by [InsP_3_]. Consequently, the correct classification of a Ca^2+^ time series implicitly constrains the governing parameters to a bounded region of this landscape, even without explicitly recovering their values. This observation establishes the present classification framework as a necessary and principled stepping stone towards full quantitative parameter inference. The natural extension of this work is the adoption of simulation-based inference (SBI) via neural posterior estimation [99,100], which would learn the full posterior distribution P(θ∣x) over biologically meaningful model parameters $\theta =\{[{\text{InsP}}_{3}],\;...\}$ conditioned on an observed Ca^2+^ time series x. Such an approach would enable the direct recovery of hidden physiological parameters from noisy experimental fluorescence traces, without requiring manual tuning or prior knowledge of the underlying dynamical regime.

## Methods

### Theoretical nonlinear model of intracellular calcium ion (Ca^2+^) Oscillations

We explain the mechanism of intracellular Ca^2+^ oscillations (panel (a) of Fig 1) as follows. Consider a cell of system size represented by *V*. When an agonist attaches to the plasma membrane receptor, it triggers the synthesis of InsP_3_ (denoted by *Z*), another intracellular second messenger. InsP_3_ binds to receptors on the endoplasmic reticulum membrane, initiating Ca^2+^-release from the internal pool (denoted by *Y*) into the cytosol through the InsP_3_ receptor/Ca^2+^ channel (IP_3_R channel). This cytosolic Ca^2+^ (denoted by *X*) further activates its own release through the IP_3_R channel, a phenomenon known as Ca^2+^-induced Ca^2+^-release (CICR), indicating an autocatalytic process that generates intracellular Ca^2+^ oscillations. Stimulation of InsP_3_ 3-kinase activity is achieved through a Ca^2+^/calmodulin complex. Ca^2+^-activated InsP_3_ degradation occurs. In the figure, β represents the degree of cell stimulation by an agonist. Bold arrows encompass stimulus-induced Ca^2+^ influx, Ca^2+^ released from the pool, and Ca^2+^ exchange with the extracellular medium.

The intracellular Ca^2+^ oscillations can be described by a nonlinear theoretical model that couples free Ca^2+^ in the cytosol along with those of Ca^2+^ stored in the internal pool and InsP_3_. Suppose variables *X*, *Y*, and *Z* represent the populations of cytosolic Ca^2+^, Ca^2+^ stored, and InsP_3_, respectively. Then *x* = *X*/*V*, *y* = *Y*/*V*, and *z* = *Z*/*V* represent their respective concentrations. The time-evolution of these concentrations governs the complex dynamics of intracellular Ca^2+^ [24] as,


$$\begin{array}{cc} \frac{dx}{dt} & =V_{0}+V_{1}\beta -V_{2}+V_{3}+k_{f}y-kx,\\ \frac{dy}{dt} & =V_{2}-V_{3}-k_{f}y,\\ \frac{dz}{dt} & =\beta V_{4}-V_{5}-\epsilon z, \end{array}$$ (4)

where


$$V_{2}=V_{M2}\frac{x^{2}}{k_{2}^{2}+x^{2}},$$

$$V_{3}=V_{M3}\frac{x^{m}}{k_{x}^{m}+x^{m}}\frac{y^{2}}{k_{y}^{2}+y^{2}}\frac{z^{4}}{k_{z}^{4}+z^{4}},$$

$$V_{5}=V_{M5}\frac{z^{p}}{k_{5}^{p}+z^{p}}\frac{x^{n}}{k_{d}^{n}+x^{n}}.$$

A detailed breakdown of the terms involved in Eq. (4) is as follows: *V*_0_ denotes the constant Ca^2+^ supply from the extracellular medium. *V*_1_ represents the maximum rate of stimulus-activated Ca^2+^ entry from the extracellular medium. The rate $V_{2}\;(V_{3})$ corresponds to Ca^2+^ pumping from the cytosol into the internal pool (release of Ca^2+^ from the internal pool to the cytosol). $V_{M2}$ and $V_{M3}$ denote their maximum values. Parameters $k_{2},\;k_{y},\;k_{x}$, and $k_{z}$ represent the threshold values for pumping, release, and activation of release by Ca^2+^ and InsP_3_, respectively. *V*_2_ is solely a function of the cytosolic Ca^2+^ concentration (*x*), whereas *V*_3_ depends on all the three concentrations *x*, *y*, and *z*. The rate constant $k_{f}$ measures the passive, linear leak of *y* into *x*, and *k* signifies the linear transport of cytosolic Ca^2+^ into the extracellular medium. *V*_4_ denotes the maximum rate of stimulus-induced InsP_3_ synthesis, and *V*_5_ represents the phosphorylation rate of InsP_3_ by the 3-kinase, an InsP_3_ metabolising enzyme. The decrease of InsP_3_ is driven by its hydrolysis by calcium-dependent 3-kinase. *k*_5_ denotes the half-saturation constant. Stimulation of InsP_3_ 3-kinase activity (through a Ca^2+^/calmodulin complex) is represented by a Hill-form term with $k_{d}$ as the threshold level of Ca^2+^. The term −ϵZ accounts for the metabolism of InsP_3_ by 5-phosphatase, independent of Ca^2+^. Additionally, cooperative processes in Ca^2+^ release from internal stores into the cytosol and phosphorylation of InsP_3_ by 3-kinase are reflected in *V*_3_ and *V*_5_, incorporating Hill-coefficients *m*, *n* and *p*. A value of *p* > 1 indicates the presence of cooperativity in 3-kinase kinetics, while *p* = 1 indicates its absence [24].

By adjusting the values of the rate constants and other model parameters in Eq. (4), concentrations *x*, *y*, and *z* exhibit various kinds of dynamical patterns [33].

### Chemical Langevin formalism

consider a well-stirred chemically reacting system of volume *V* that is maintained at a fixed temperature *T*. Suppose the system consists of *N* molecular species whose molecular populations is represented by $X_{1}(t),X_{2}(t),\ldots ,X_{N}(t)$. Imagine the *N* species are now interacting through *M* chemical reactions, where each reaction channel is represented by: ${\text{a}}_{1j}{\text{X}}_{1}+{\text{a}}_{2j}{\text{X}}_{2}+\ldots +{\text{a}}_{\text{Nj}}{\text{X}}_{\text{N}}\overset{{\text{k}}_{j}\;}{\to }{\text{b}}_{1j}{\text{X}}_{1}+{\text{b}}_{2j}{\text{X}}_{2}+\ldots +{\text{b}}_{\text{Nj}}{\text{X}}_{\text{N}}$, where $k_{j}\;(\;j=1,2,\ldots ,M)$ denotes the classical rate constant of the $j^{th}$ reaction. The co-efficients $\left\{a_{1},a_{2},\ldots ,a_{N}\right\}$ and $\left\{b_{1},b_{2},\ldots b_{N}\right\}$ denote the numbers of reactant and product molecules, respectively. Denoting $\text{X}(t)=[X_{1}(t),X_{2}(t),\ldots ,X_{N}(t){]}^{T}$, with T representing transpose, as the state vector of molecular populations in the system, the probability of a reaction $R_{j}$ occurring within *V* in the next infinitesimal time interval (*t*,*t* + *dt*) is given by $a_{j}(\text{X})dt$, where $a_{j}(\text{X})$ is the propensity function [71] for $R_{j}$, where it is expressed as $a_{j}(\text{X})=c_{j}h_{j}(\text{X})$ [71,101]. Here $h_{j}(\text{X})$ accounts for possible combinations of molecules for reaction $R_{j}$. The stochastic rate constant $c_{j}$ is related to the classical rate constant $k_{j}$ by the relation $c_{j}=k_{j}V^{1-\nu_{j}}$, with $\nu_{j}$ being the stoichiometric coefficient in reaction $R_{j}$. We now describe the chemical Langevin equation (CLE) following Gillespie’s formalism [71]. For an arbitrary *t*ime in*t*erval *dt* > 0, if $\Lambda_{j}[\text{X}(t),dt]$ describes the number of $R_{j}$ reactions occurring in the subsequent time interval (*t*,*t* + *dt*), then the molecular population $X_{i}$ at time *t* + *dt* is


$$X_{i}(t+dt)=X_{i}(t)+\sum_{j=1}^{M}\Lambda_{j}[\text{X}(t),dt]\;\nu_{ji},$$ (5)

where i=1,2,…,N, and $\nu_{ji}$ is the change in $X_{i}$ due to $R_{j}$. However, to determine $\Lambda_{j}[\text{X}(t),dt]$ is a challenge. To approximate the function $\Lambda_{j}$, two important conditions are imposed on Eq. (5). Firstly, we require the time interval dt→small such that the reaction events occurring during (*t*,*t* + *dt*) do not significantly change the propensity functions, i.e., $\Delta a_{j}=a_{j}(\text{X}(t^{\prime }))-a_{j}(\text{X}(t))≅0,\forall \;t^{\prime }\in (t,t+dt)$. This condition holds when the reactant populations are large (≫1), allowing each $\Lambda_{j}$ to be approximated by a statistically independent Poisson random variable, i.e., $\Lambda_{j}\to {𝒫}_{j}(a_{j}(\text{X}),dt)$. Secondly, we require dt→large enough such that the number of $R_{j}$ reaction events during (*t*,*t* + *dt*) is far greater *t*han uni*t*y, i.e., $a_{j}(\text{X})dt\gg 1$. This enables each ${𝒫}_{j}(a_{j}(\text{X}),dt)$ to be approximated by a normal random variable such that ${𝒫}_{j}(a_{j}(\text{X}),dt)\to {𝒩}_{j}(a_{j}(\text{X})dt,a_{j}(\text{X})dt)$. These two conditions are applied simultaneously in the limit of a large molecular population, rendering *dt macroscopically infinitesimal* [71].

Representing μ and σ as the mean and standard deviation, respectively, and using $𝒩(\mu ,\sigma^{2})=\mu +\sigma 𝒩(0,1)$ [70], we arrive at


$$X_{i}(t+dt)=X_{i}(t)+\sum_{j=1}^{M}\nu_{ji}\;a_{j}(\text{X})dt+\sum_{j=1}^{M}\nu_{ji}\;[a_{j}(\text{X})dt{]}^{1/2}\;{𝒩}_{j}(0,1),$$ (6)

which is the standard-form Langevin equation [70,71].

Rearranging Eq. (6) yields


$$\frac{X_{i}(t+dt)-X_{i}(t)}{dt}=\sum_{j=1}^{M}\nu_{ji}\;a_{j}(\text{X})+\sum_{j=1}^{M}\nu_{ji}\;a_{j}^{1/2}(\text{X}){𝒩}_{j}(0,1)dt^{-1/2},$$ (7)

where $𝒩(0,1)dt^{-1/2}=𝒩(0,1/dt)$ [70]. Taking the limit dt→0, the CLE is obtained as [70,71]:


$$\frac{dX_{i}(t)}{dt}=\sum_{j=1}^{M}\nu_{ji}\;a_{j}(\text{X})+\sum_{j=1}^{M}\nu_{ji}\;a_{j}^{1/2}(\text{X})\;\xi_{j}(t),$$ (8)

with $\xi_{j}(t)=\lim_{dt\to 0}𝒩(0,1/dt)$ [70,71] as temporally uncorrelated, statistically independent Gaussian white noises. The first term of the right-hand side represents the deterministic part, whereas the second term denotes the stochastic term. The CLE (8) therefore accounts for both deterministic and stochastic components in the dynamics of a chemically reacting system.

In Eq. (8), the drift and diffusion components both follow from the Poisson random variable ${𝒫}_{j}$ [71]. Thus, the ratio of the random to the deterministic component is given by $a_{j}^{-1/2}(\text{X})$. For a Poisson distribution, we have the standard deviation $\sigma =\sqrt{\mu }$. Assuming the average number of molecules μ is proportional to the system size *V*, we get $\sigma \propto \sqrt{V}$. Consequently, the magnitude of intrinsic fluctuations in molecular populations, given by (1/σ), scales as $\left(1/\sqrt{V}\right)$. Thus, for stochastic chemical reactions described by Poisson random variable ${𝒫}_{j}$, intrinsic fluctuations in molecular populations scale as the inverse square root of the system size, i.e., $\frac{1}{\sqrt{V}}$ [71].

### Stochastic modeling with chemical Langevin equation

Suppose *x*(*t*), *y*(*t*), *z*(*t*) represent the concentrations of cytosolic Ca^2+^, Ca^2+^ stored, and InsP_3_, respectively. Let the state vector of concentrations is $s=s(t)=[x(t),\;y(t),\;z(t){]}^{T}$. The evolution equation for the state, given previously in Eq. 4, can be re-written in a compact form as


$$\frac{ds}{dt}=F(x,y,z),$$ (9)

where


$$F(x,y,z)=[\begin{array}{c} V_{0}+V_{1}\beta -V_{2}+V_{3}+k_{f}y-kx\\ V_{2}-V_{3}-k_{f}y\\ \beta V_{4}-V_{5}-\epsilon z \end{array}].$$

The system of ODEs in Eq. (9) can be reformulated as reaction channels [33], highlighting the variation in populations *X*, *Y*, and *Z* as follows:

**Table:** 

Reaction channels	Propensity functions
X→X+1	*V V*_0_
X→X+1	$VV_{1}\beta$
X→X−1	*V V*_2_
X→X+1	*V V*_3_
X→X+1	$Vk_{f}y$
X→X−1	*V kx*
Y→Y+1	*V V*_2_
Y→Y−1	*V V*_3_
Y→Y−1	$Vk_{f}y$
Z→Z+1	$V\beta V_{4}$
Z→Z−1	*V V*_5_
Z→Z−1	Vϵz

In the second column, the propensity function for each reaction $R_{j}$ is calculated using Gillespie’s formalism [71,101]. The changes in the state *s* thus correspond to stochastic events involving the births and deaths of molecular species labeled as *X*, *Y*, and *Z*. These events inherently introduce randomness within the populations of these molecular species. Subsequently, we proceed to compute the CLE pertaining to the intracellular Ca^2+^ oscillation model (9) as follows:


$$\frac{ds}{dt}=F(x,y,z)+\frac{1}{\sqrt{V}}\;G(x,y,z),$$ (10)

where *V* denotes the system size, and *G*(*x*,*y*,*z*) is:


$$G(x,y,z)=[\begin{array}{c} \sqrt{V_{0}}\xi_{1}+\sqrt{V_{1}\beta }\xi_{2}-\sqrt{V_{2}}\xi_{3}+\sqrt{V_{3}}\xi_{4}+\sqrt{k_{f}y}\xi_{5}-\sqrt{kx}\xi_{6}\\ \sqrt{V_{2}}\xi_{7}-\sqrt{V_{3}}\xi_{8}-\sqrt{k_{f}y}\xi_{9}\\ \sqrt{V_{4}\beta }\xi_{10}-\sqrt{V_{5}}\xi_{11}-\sqrt{\epsilon z}\xi_{12} \end{array}].$$ (11)

The stochastic differential equation (10) reduces to the deterministic equation (9), describing the mean-field behavior of the system, when V→∞.

To simulate the intracellular Ca^2+^ dynamics, we numerically solve the CLE (1) using the *Euler-Maruyama* method, where the simulation is performed with a fixed time-step of 10^−6^ min. The volume parameter *V* is given in cubic micrometers ($\mu {\text{m}}^{3}$) [33].

### Large Kernel Convolutional Neural Network (LKCNN)

For our Ca^2+^ dynamics classification task, the LKCNN architecture proceeds in such a way that the large kernel slides across the input time series to extract temporal features through successive convolutional layers and the extracted features are then flattened and processed through fully connected layers to produce the final classification output (as illustrated in Fig 1(c)). This model architecture enables the network to capture both local temporal patterns and long-range dependencies.

The LKCNN classifier, we use a feed-forward architecture motivated by earlier approaches used for the classification of dynamical states [65,66]. The architectural details of the LKCNN utilized in our work are as follows:

- Convolutional Layer 1: 16 filters, kernel size *k*, stride 2 → Max Pooling (size 2)
- Convolutional Layer 2: 32 filters, kernel size *k*, stride 2 → Max Pooling (size 2)
- Dropout (rate 0.5) → Flatten
- Fully Connected Layers: 64 units → Dropout (rate 0.5) → 32 units → Output

The neural network uses ReLU activation functions for all hidden layers and is trained by minimizing the categorical cross-entropy loss function [102] ℒ given by:


$$ℒ=-\frac{1}{N}\sum_{i=1}^{N}\sum_{j=1}^{C}y_{i,j}\log({\hat{y}}_{i,j}),$$

where *N* is the batch size, *C* is the number of classes, and $y_{i,j}$ is the ground-truth label. The term ${\hat{y}}_{i,j}$ represents the model’s predicted probability, calculated by applying the softmax function [102] to the logits $z_{i,j}$ from the final output layer:


$${\hat{y}}_{i,j}=\frac{\exp(z_{i,j})}{\sum_{k=1}^{C}\exp(z_{i,k})}.$$

For optimization, we employ the Adam algorithm with a learning rate of $\alpha =10^{-4}$ and a batch size of *N* = 32. The training continues for at most 2,000 epochs, incorporating an early stopping mechanism with a patience of 100 on the validation accuracy to prevent overfitting and select the best-performing model. To determine the optimal model configuration and ensure the robustness of our results, this entire procedure is systematically repeated for various kernel sizes, k∈{4,5,6,…,100}, and across 20 different random seeds. This rigorous exercise has identified an optimal kernel size of 28. The confusion matrices and final results presented in the main text are derived from the model trained using this optimal configuration.

### Parameters used for synthetic data generation

See Table 1

### Performance of optimized classifier

See Table 2 and 3

## Supporting information

S1 Fig Cycle-to-cycle amplitude variability of noiseless Period-2 trajectories and its relation to Period-4 misclassification. Each point represents one noiseless synthetic Period-2 (P-2) test trajectory. For each trajectory, we first detect the local minima and local maxima over the finite observation window, and then quantify how much their amplitudes vary from cycle to cycle. The x-axis shows the relative variability of local-minimum amplitudes across cycles, and the y-axis shows the relative variability of local-maximum amplitudes across cycles; in both cases, the variability is normalized by the total amplitude range of the corresponding trajectory so that values can be compared across samples. Thus, points near the origin correspond to Period-2 trajectories whose successive minima and maxima are highly consistent from cycle to cycle, whereas points farther from the origin indicate trajectories with larger cycle-to-cycle amplitude fluctuations. Grey points denote correctly classified samples (P-2 → P-2), and red points denote samples misclassified as Period-4 (P-2 → P-4). The red points are concentrated in the high-variability region, indicating that the observed P-2 → P-4 confusion is associated with Period-2 trajectories whose cycle-to-cycle amplitude pattern is less regular. In a finite-length time window, such irregular amplitude alternation can make a Period-2 trajectory appear more similar to a higher-period pattern, thereby increasing the likelihood of misclassification as Period-4.
(PNG)

S2 Fig Confusion matrices for conventional FFT-based baselines (SVM and Random Forest). Row-normalized confusion matrices for (a) a linear-kernel SVM and (b) a Random Forest trained on FFT-transformed features of the trajectories. For each method, classification performance (%) is shown on the noiseless synthetic test set (top), the noisy synthetic test set (center), and the experimental dataset (bottom). Synthetic evaluations use the 8-class label set, whereas the experimental evaluation employs the 3-label mapping (Bursting, Steady State, Others) used for comparison with human annotation.
(PNG)

S3 Fig Representative examples of spike-like corruption in synthetic Ca^2+^ trajectories. Example trajectories from bursting, chaotic, and period-4 states (at *V* = 10^5^) after applying sparse, high-amplitude spike perturbations at randomly selected time points, that is used for probing robustness against outlier-like artifacts.
(PNG)
