# Supplementary figures and images for: Stochastic intracellular calcium dynamics show preserved structures identified by deep learning classification
Source: PLoS Comput Biol. 2026 Apr 29;22(4):e1014240. doi: 10.1371/journal.pcbi.1014240 (PMC13143184; doi:10.1371/journal.pcbi.1014240)

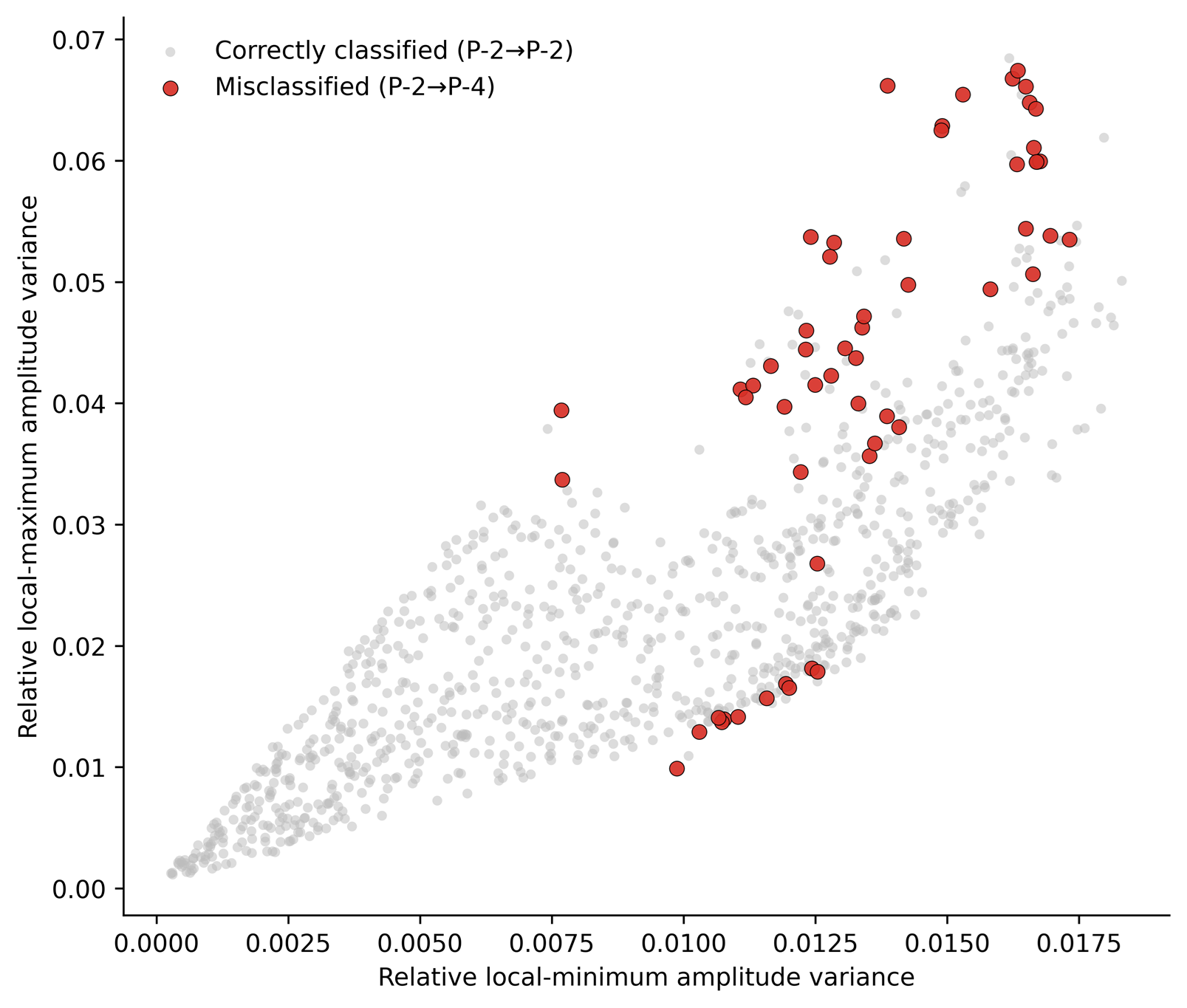

Supplement: S1 Fig — Each point represents one noiseless synthetic Period-2 (P-2) test trajectory. For each trajectory, we first detect the local minima and local maxima over the finite observation window, and then quantify how much their amplitudes vary from cycle to cycle. The x-axis shows the relative variability of local-minimum amplitudes across cycles, and the y-axis shows the relative variability of local-maximum amplitudes across cycles; in both cases, the variability is normalized by the total amplitude range of the corresponding trajectory so that values can be compared across samples. Thus, points near the origin correspond to Period-2 trajectories whose successive minima and maxima are highly consistent from cycle to cycle, whereas points farther from the origin indicate trajectories with larger cycle-to-cycle amplitude fluctuations. Grey points denote correctly classified samples (P-2 → P-2), and red points denote samples misclassified as Period-4 (P-2 → P-4). The red points are concentrated in the high-variability region, indicating that the observed P-2 → P-4 confusion is associated with Period-2 trajectories whose cycle-to-cycle amplitude pattern is less regular. In a finite-length time window, such irregular amplitude alternation can make a Period-2 trajectory appear more similar to a higher-period pattern, thereby increasing the likelihood of misclassification as Period-4. (PNG) [file pcbi.1014240.s001.png]

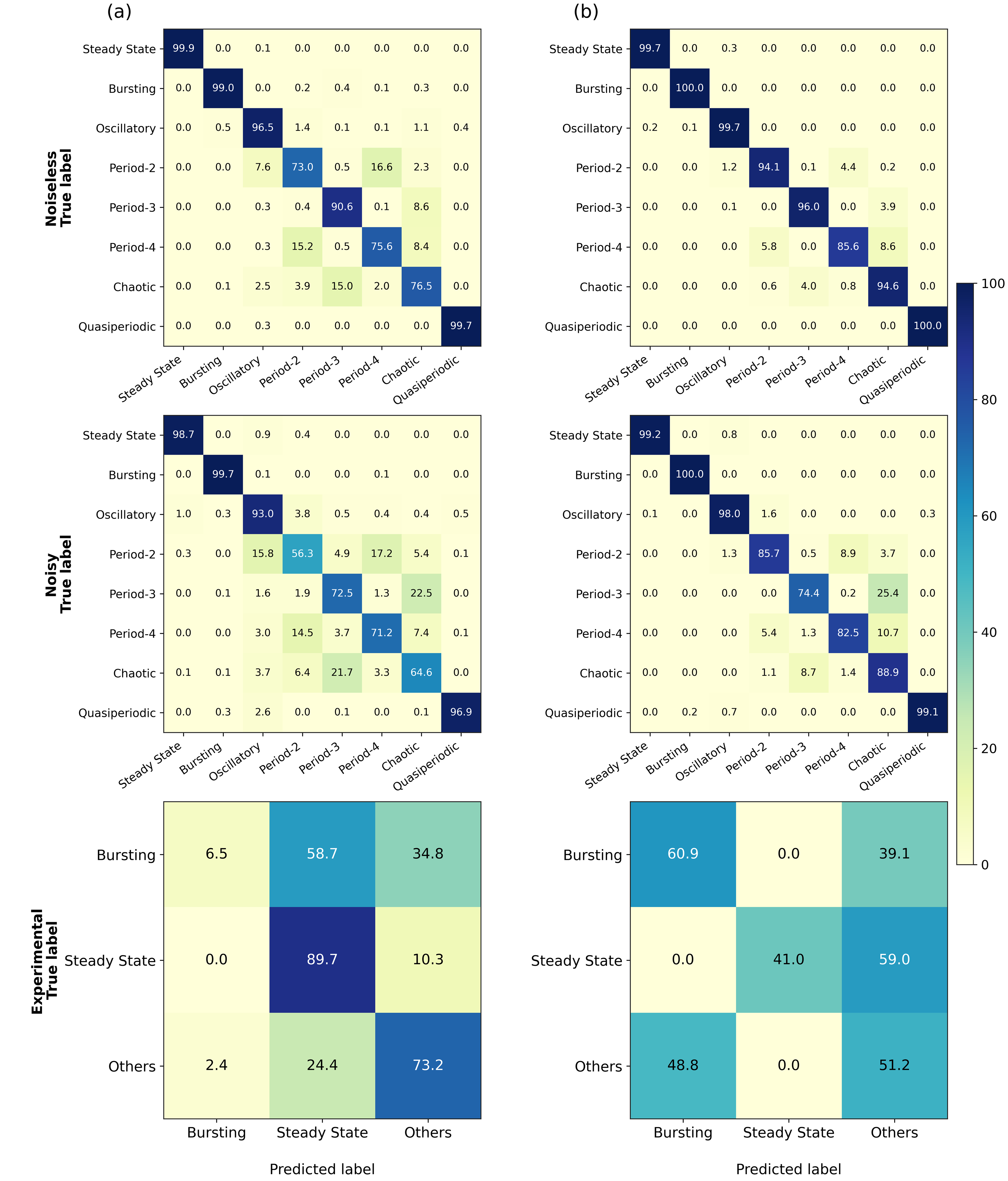

Supplement: S2 Fig — Row-normalized confusion matrices for (a) a linear-kernel SVM and (b) a Random Forest trained on FFT-transformed features of the trajectories. For each method, classification performance (%) is shown on the noiseless synthetic test set (top), the noisy synthetic test set (center), and the experimental dataset (bottom). Synthetic evaluations use the 8-class label set, whereas the experimental evaluation employs the 3-label mapping (Bursting, Steady State, Others) used for comparison with human annotation. (PNG) [file pcbi.1014240.s002.png]

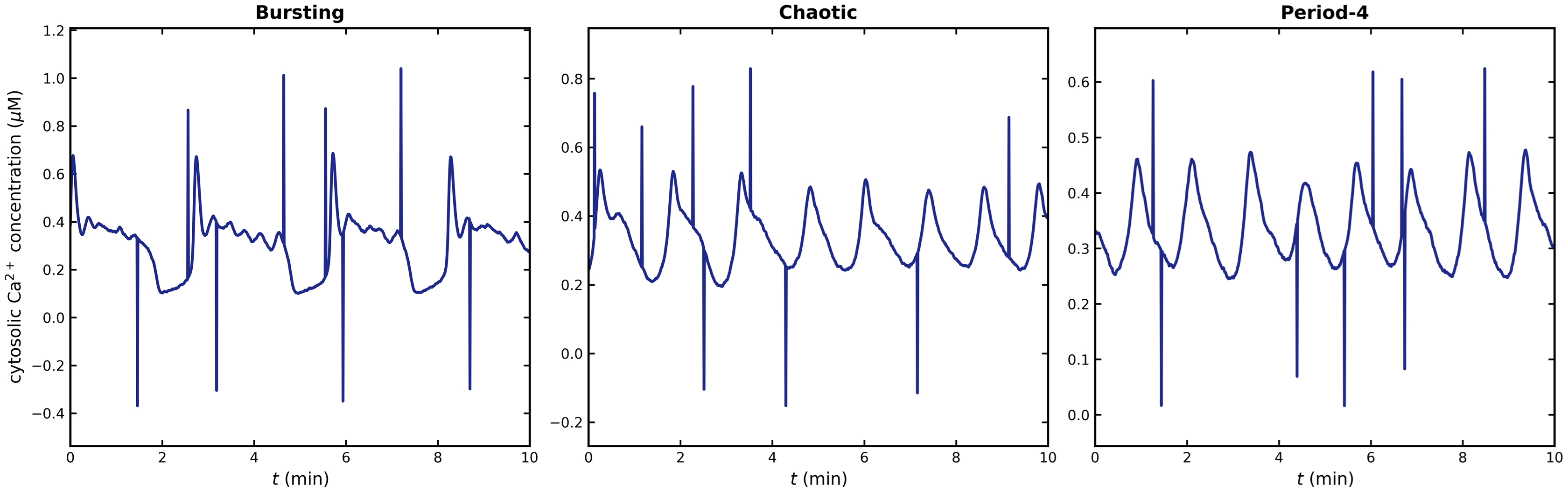

Supplement: S3 Fig — Example trajectories from bursting, chaotic, and period-4 states (at V = 105) after applying sparse, high-amplitude spike perturbations at randomly selected time points, that is used for probing robustness against outlier-like artifacts. (PNG) [file pcbi.1014240.s003.png]
